# *Pseudomonas* sp. LZ-Q continuously degrades phenanthrene under hypersaline and hyperalkaline condition in a membrane bioreactor system

**DOI:** 10.1007/s41048-016-0018-3

**Published:** 2016-03-17

**Authors:** Yiming Jiang, Haiying Huang, Mengru Wu, Xuan Yu, Yong Chen, Pu Liu, Xiangkai Li

**Affiliations:** Ministry of Education Key Laboratory of Cell Activities and Stress Adaptations, School of Life Science, Lanzhou University, Lanzhou, 730000 China; State Key Laboratory of Microbial Resources, Institute of Microbiology, Chinese Academy of Sciences, Beijing, 100101 China; Department of Development Biology Sciences, School of Life Science, Lanzhou University, Lanzhou, 730000 China

**Keywords:** *Pseudomonas* sp. LZ-Q, Phenanthrene degradation, Immobilization microorganisms, Hypersaline and hyperalkaline wastewater, Membrane bioreactor (MBR)

## Abstract

****Graphical Abstract**:**

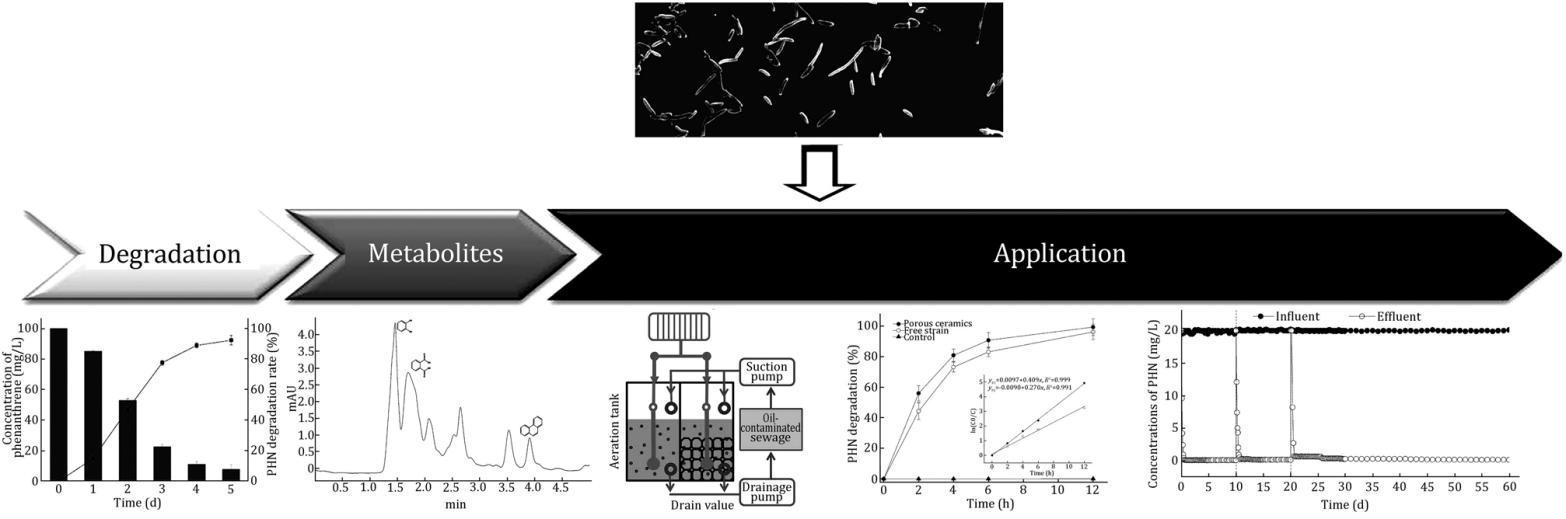

**Abstract:**

Phenanthrene is one of the most recalcitrant components of crude oil-contaminated wastewater. An efficient phenanthrene-degrading bacterium *Pseudomonas* sp. strain named LZ-Q was isolated from oil-contaminated soil near the sewage outlet of a petrochemical company. *Pseudomonas* sp. LZ-Q is able to degrade 1000 mg/L phenanthrene in Bushnell-Hass mineral salt medium. It also degrades other polycyclic aromatic hydrocarbons such as naphthalene, anthracene, pyrene, petrol, and diesel at broad ranges of salinities of 5 g/L to 75 g/L, pHs of 5.0–10.0, and temperatures of 10–42 °C. Therefore, *Pseudomonas* sp. LZ-Q could be a good candidate for remediation of polycyclic aromatic hydrocarbon (PAH)-contaminated wastewater. A membrane bioreactor (MBR) was applied to investigate the remediation ability of the strain LZ-Q. Wastewater containing phenanthrene with pH of 8, salinity of 35 g/L, and COD of 500 mg/L was continuously added to the system (HRT = 3 h). Results showed that *Pseudomonas* sp. LZ-Q is capable of degrading 96% of 20 mg/L phenanthrene and 94% of 500 mg/L COD for 60 days in a continuous mode. These results showed that the MBR system with strain LZ-Q might be a good approach for PAHs’ remediation in industrial wastewaters.

## INTRODUCTION

Nowadays, water pollution is becoming an increasingly concerned environmental problem (Schwarzenbach et al. 2010). In the northwest of China, water pollution events occur frequently. Among the refractory organics causing water pollution events, polycyclic aromatic hydrocarbons (PAHs) are considered to be the most environmentally significant and hazardous to human health (Wang et al. 2013). PAHs are a group of organic chemicals consisting of two or more fused benzene rings that are in linear, angular, and cluster arrangements (Bamforth and Singleton 2005). Most of the PAHs are toxic, mutagenic, carcinogenic, and recalcitrant (Wu et al. 2010; Patel et al. 2012). PAHs released into the environment would cause serious risks to natural environment, fishery, agriculture and human health (Wang et al. 2013). Therefore, controlling PAHs pollution is an urgent task in water protection.

Phenanthrene (PHN) identified as one of the priority pollutants is a typical PAH and some of its derivatives are carcinogenic (Jerina et al. 2012). Microbes are able to degrade PHN and provide an ideal bioremediation approach. For example, *Pseudomonas stutzeri* ZP2 can degrade more than 90% of PHN at 1000 ppm in 6 days (Zhao et al. 2009). *Pseudomonas* sp. JM2 isolated from active sewage sludge of a chemical plant removes 50 mg/L PHN within 4 days (Ma et al. 2012). However, successful applications of using microbes to remediate PHN in industrial wastewater are still scarce (Lefebvre and Moletta 2006). Industrial wastewater with wide ranges of pHs and hypersalinities inhibits microbial respiration rates, reduces enzymes’ activity, and elevates osmotic pressure of cells. Many of the known PHN-degrading bacteria cannot survive well under such condition and function properly (Kunst and Rapoport 1995; Metcalf 2003). Therefore, searching for a PHN degrading strain with survival ability in industrial wastewater is critical for applications of PHN’s remediation.

Membrane bioreactor (MBR) is proven to be a good method for wastewater treatment and attracts extensive attentions, because MBR is characterized with high pollutant removal efficiency. Removal of PAHs by MBR has been studied (González et al. 2012). However, membrane fouling is one significant limitation when using MBR to treat wastewater (Tang et al. 2010; Zhang et al. 2011). Previous studies have demonstrated that microorganism immobilization technology (MIT) can improve the effectiveness of sewage treatment and enable cells to separate from aqueous solution easily and reduce the membrane fouling. MIT has been widely applied in industrial operation and works efficiently (Juang et al. 2008; Bai et al. 2009; Ting and Sun 2000; Yan and Viraraghavan 2001). Immobilizing microbiota B500 on macro-porous carriers enhances the removal efficiency for contaminants in wastewater (Park et al. 2005). Iron-oxidizing bacteria immobilized onto polyurethane foam decrease the risk of membrane fouling and increase the efficiency of pollutants’ degradation (Zhou et al. 2008).

In this study, a bacterial strain LZ-Q utilizing PHN as the sole carbon source was isolated from petroleum-contaminated soil. Ceramics are used as carriers of strain LZ-Q in MBR system. Strain LZ-Q degraded PHN efficiently and this MBR with immobilized strain LZ-Q cells showed ability of degradation of PHN in artificial petrochemical wastewater in long period.

## RESULTS AND DISCUSSION

### Isolation and characterizations of strain LZ-Q

**Fig. 1:**
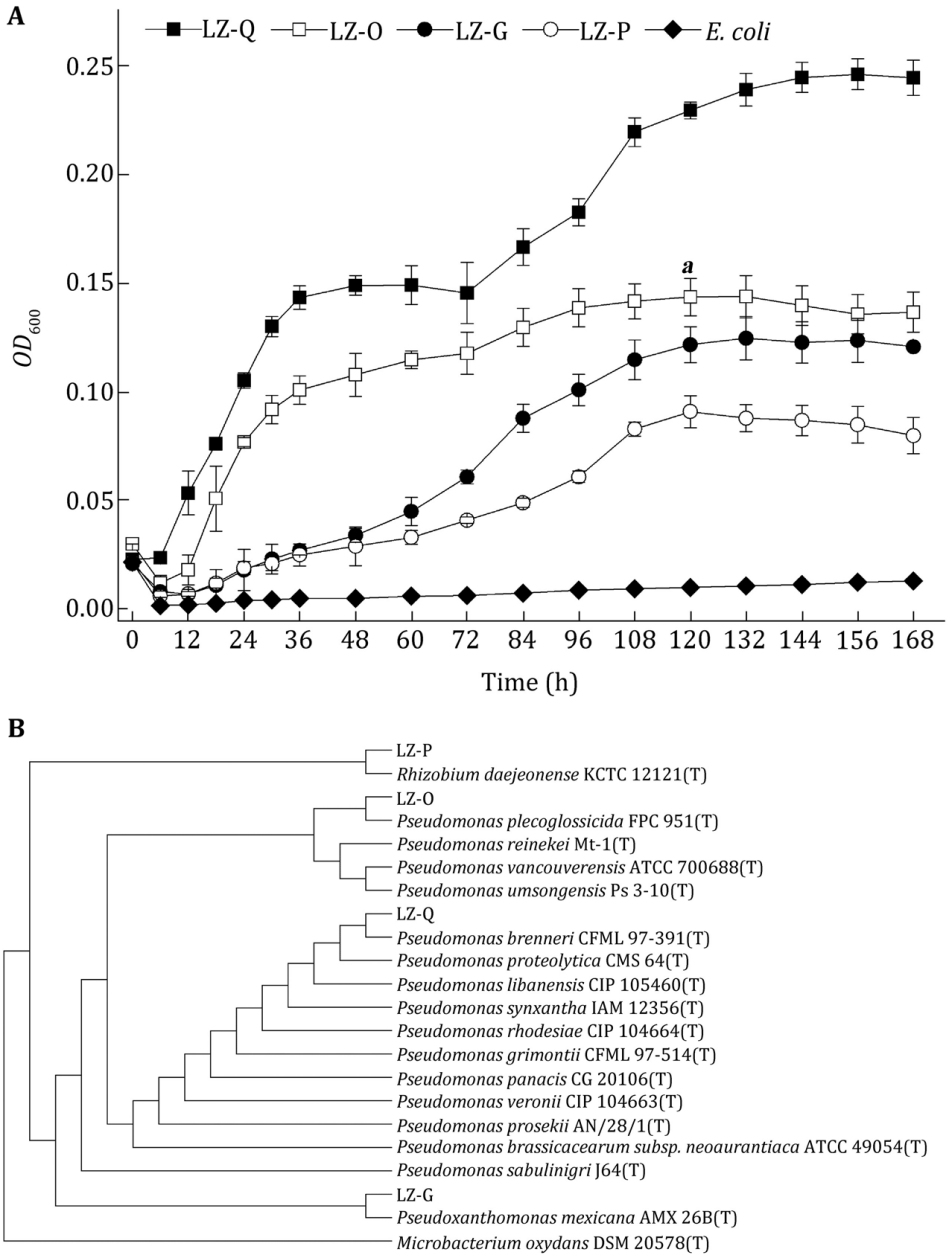
**A** Growth curves of isolated strains and *E. coli*. **B** Phylogenetic tree based on 16S rRNA gene sequence showing the relationship between corresponding sequences of the genus *Pseudomonas* genus

Four bacterial strains with the ability of phenanthrene degradation were isolated from petrochemical-contaminated soils in Lanzhou reach of the Yellow River, China. All four strains were gram-negative and aerobic. By comparison of 16S rRNA gene sequences, LZ-Q (GenBank No.: KR140091, CCTCC No.: M2015564), LZ-O (GenBank No.: KR140089), and LZ-G (GenBank No.: KR140088) were closely related to *Pseudomonas* spp. and LZ-P (GenBank No.: KR140090) was related to *Rhizobium* sp. Growths of the isolated strains using phenanthrene (1000 mg/L) as the sole carbon source were determined at pH 7, 180 r/min, and 28 °C. All isolated strains can grow under such condition and strain LZ-Q reached highest optical density (*OD*_600nm_) of 0.25 after 168 h incubation (Fig. 1A). This result suggests that all four strains can degrade PHN, and strain LZ-Q was chosen for further study as it showed higher growth when using PHN as sole carbon source. The strain LZ-Q was short rod-shaped bacterium. The colonies of strain LZ-Q were mostly small, opaque, circular or irregular oval-shaped and oyster white-colored with moist and luster surface. ViTek phenotype analysis showed that strain LZ-Q was 95% closely related to *Pseudomonas fluorescens* (Table 1). A phylogenetic tree based on neighbor-joining algorithm demonstrated that LZ-Q clustered with *P. brenneri,* which falls within the *P. fluorescens* group, at a bootstrap value of 75% (Fig. 1B). Our data also showed that strain LZ-Q degrades various PAHs, petrol, and diesel, suggesting it might be an ideal strain for PAH bioremediation (Table 1).

**Table 1:** ViTek report of strain LZ-Q

Biochemical details																	
2	APPA^−^	−	3	ADO	−	4	PyrA	+	5	IARL	−	7	dCEL	−	9	BGAL	−
10	H_2_S	−	11	BNAG	−	12	AGLTp	−	13	dGLU	+	14	GGT	−	15	OFF	−
17	BGLU	−	18	dMAL	−	19	dMAN	−	20	dMNE	+	21	BXYL	−	22	BAlap	−
23	ProA^−^	+	26	LIP	−	27	PLE	−	29	TyrA	+	31	URE	−	32	dSOR	−
33	SAC-	−	34	dTAG	−	35	dTRE	+	36	CIT	+	37	MNT	−	39	5 KG	−
40	ILATk-	−	41	AGLU	−	42	SUCT	+	43	NAGA	−	44	AGAL	−	45	PHOS	−
46	GlyA-	−	47	ODC	−	48	LDC	−	53	IHISa	−	56	CMT	−	57	BGUR	−
58	O129R	+	59	GGAA	−	61	IML Ta	+	62	ELLM	−	64	lLATa	−			

“+” and “−” represent whether the strain can utilize the substrate or not

### Characterization of phenanthrene degradation in strain LZ-Q

**Fig. 2:**
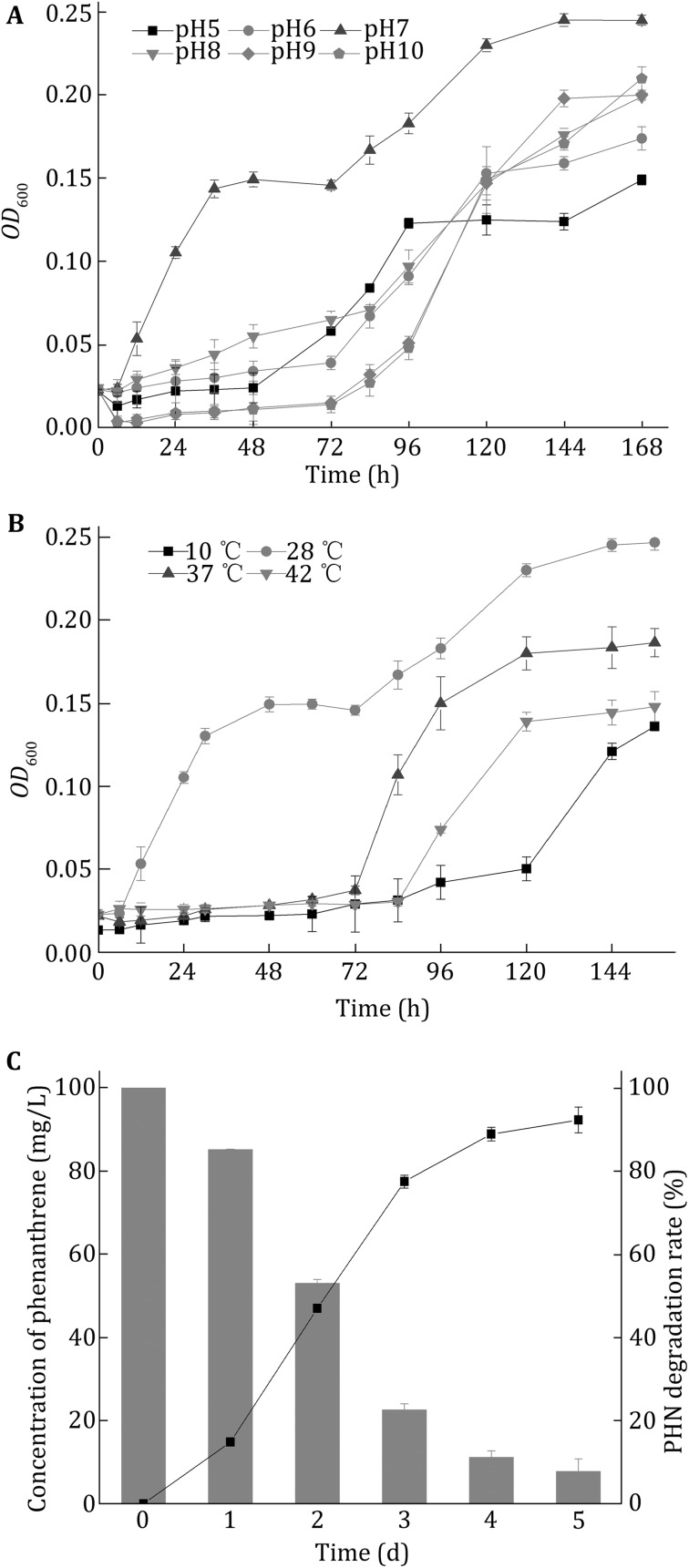
**A** Optimum pH conditions of strain LZ-Q. **B** Optimum temperature conditions of strain LZ-Q. **C** Biodegradation rate of PHN under optimum conditions

In order to determine the optimum degradation condition, strain LZ-Q was cultivated in BH medium with 100 mg/L PHN under pHs ranging from 5 to 10 and temperatures varying from 10 °C to 42 °C. Highest *OD*_600nm_ was achieved at pH 7.0 and 28 °C, which indicates that the optimum growth and PHN degrading condition for strain LZ-Q is at pH 7.0 and 28 °C (Fig. 2A, B). Under the optimum growth condition, strain LZ-Q degraded 92.27% PHN after 5 days cultivation (Fig. 2C).

Studies about *Pseudomonas* spp. which can degrade phenanthrene have been reported previously. Bacterial strain *P.* sp. Ph6-gfp isolated from clover grown in a PAH-contaminated site showed a 81.1% decrease of phenanthrene (50 mg/L) within 15 days (Sun et al. 2014), and strain *P.* stutzeri ZP2 isolated from soil in oil refinery fields in Shanghai China could reduce about 96% PHN (250 mg/L) within 6 days (Janbandhu and Fulekar 2011). In line with previous studies, strain LZ-Q that is closely related to *Pseudomonas* genus can utilize a range extension of refractory organics and degrade high concentrations of phenanthrene (Lin et al. 2014). The degradation rate is higher than *P.* sp. but lower than *P.* stutzeri ZP2 (Zhao et al. 2009; Sun et al. 2014). In addition, the results also showed that strain LZ-Q can grow at pHs of 5.0–10.0 and temperatures ranging from 10 °C to 42 °C, suggesting that it was capable of degrading PHN using phenanthrene as the sole carbon source at broad pHs and temperatures. Therefore, even though the degradation rate of PHN is not the most efficient, strain LZ-Q could degrade PHN more efficiently as the bio-mediation condition in wastewater treatment plant is variable.

**Fig. 3:**
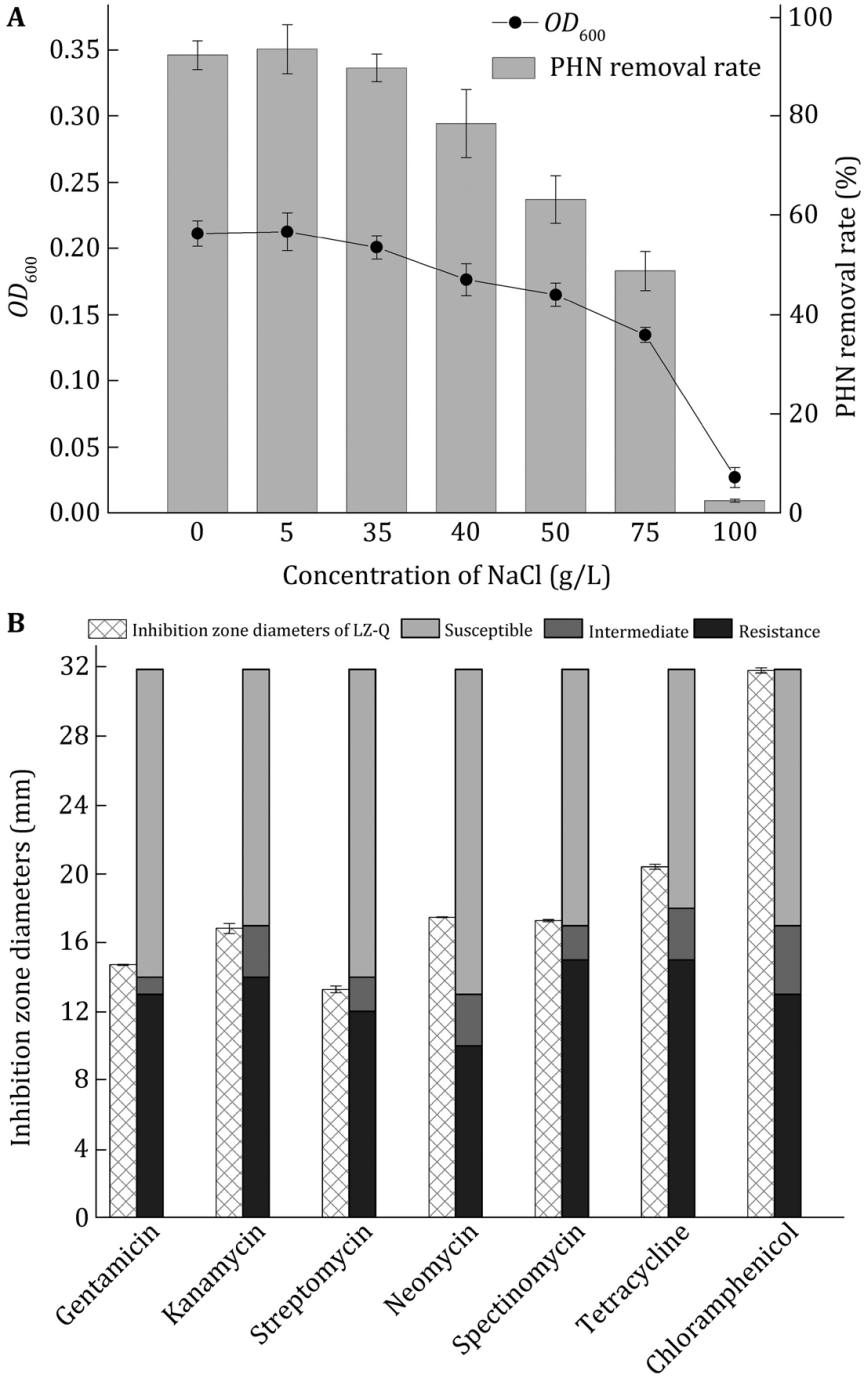
**A** *OD*_600_ and PHN degradation rate of LZ-Q under saline condition. **B** The antibiotic resistance of strain LZ-Q

Degradation of PHN is often affected by the high salinity (Haritash and Kaushik 2009). To further determine the influence of high salinity on growth and PHN degradation of strain LZ-Q, the adaptability to salinity was investigated in BH medium with the addition of different concentrations of NaCl (5, 10, 35, 40, 50, 75 and 100 g/L) after 120 h of incubation. Strain LZ-Q degraded PHN with 5 g/L to 75 g/L NaCl (Fig. 3A). These results showed that strain LZ-Q could degrade PHN efficiently under hypersaline condition. As the high salinity of industrial wastewater usually restricts microorganisms to remove pollutants, studies about bacterial strains which degraded PHN under hypersaline condition and were used in industrial wastewater plant were limited (Kunst and Rapoport 1995; Metcalf 2003). *P.* sp. BZ-3 degraded 75% phenanthrene under the 20 g/L NaCl (Lin et al. 2014). Our data showed that strain LZ-Q also remove phenanthrene contaminants under high salinity (75 g/L NaCl) and high alkalinity (pH 9) conditions efficiently. Results showed that LZ-Q’s PHN degradation ability which is 3% lower than *P.* stutzeri ZP2, is not the highest at optimum conditions. But it can degrade PHN at pHs and temperatures which other strains cannot, suggesting that strain LZ-Q is a potential strain for application.

Previous studies proposed that multi-drug-resistant strains have a higher risk of spreading antibiotic resistance genes to indigenous flora (Masakorala et al. 2013). The antibiotic sensitive tests showed strain LZ-Q was sensitive to gentamicin, neomycin, spectinomycin, tetracycline, chloramphenicol, and intermediately susceptible to streptomycin and kanamycin (Fig. 3B). These results demonstrate that strain LZ-Q displays a profile of low resistance to antibiotics. Thus, *P.* sp. LZ-Q could be a suitable bioremediation additive in in situ wastewater treatment.

All the results provide the evidence that *P.* sp. LZ-Q might be a good potential candidate for the bioremediation of phenanthrene-contaminated industrial sewage under hypersaline and hyperalkaline conditions.

### Metabolic pathways of phenanthrene degradation by strain LZ-Q

**Fig. 4:**
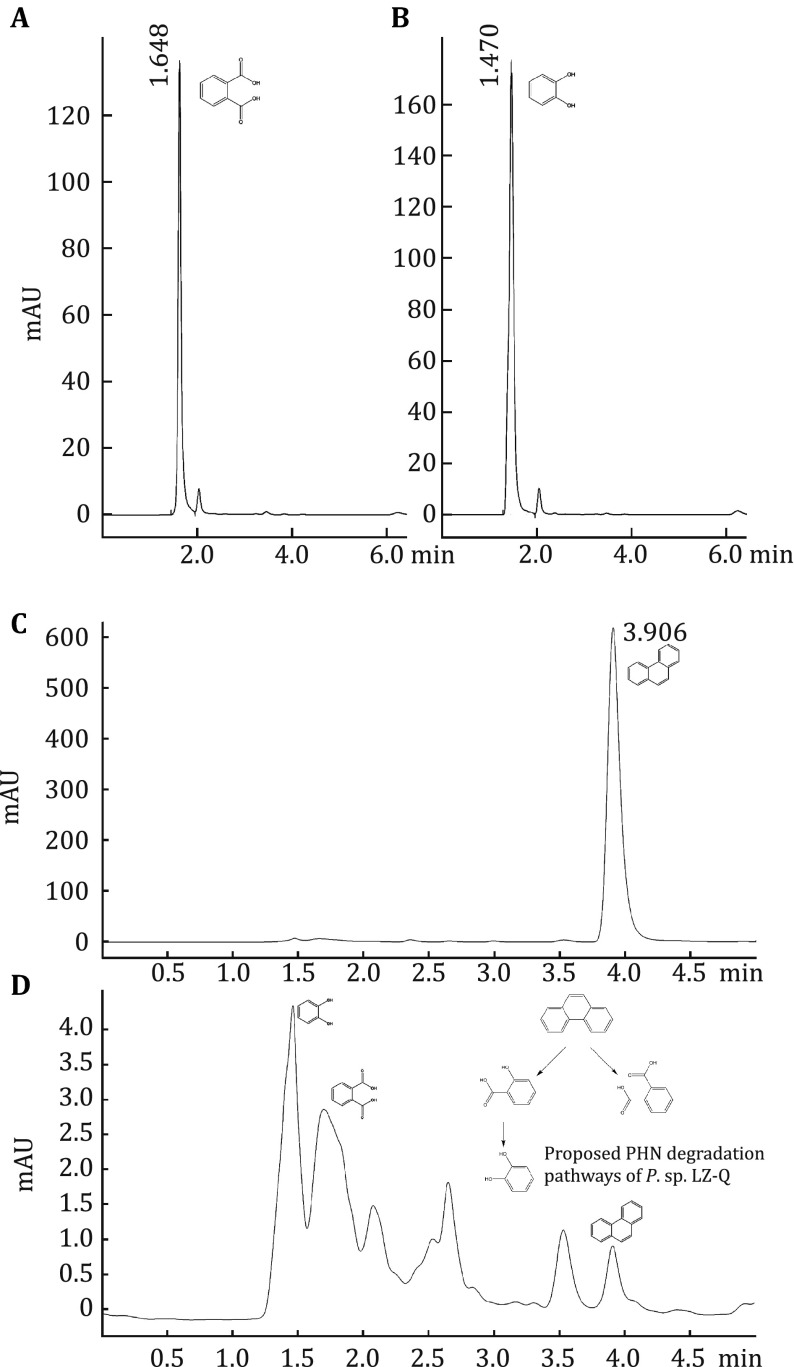
Metabolite analysis using HPLC. Standard substances of phthalic acid (**A**), catechol (**B**) and phenanthrene (**C**); **D** Metabolite analysis

Two metabolic pathways including salicylic acid pathway and phthalic acid pathway were reported to degrade phenanthrene by bacteria (Prabhu and Phale 2003). Salicylic acid and catechol are intermediates of the salicylic acid pathway, phthalic acid is generated in the phthalic acid pathway (Peng et al. 2008; Haritash and Kaushik 2009). Strain LZ-Q utilizes salicylic acid, catechol, and phthalic acid as carbon sources (Table 2), suggesting that strain LZ-Q degrades phenanthrene through both salicylic acid pathway and phthalic acid pathway. The possible pathways for PHN degradation by strain LZ-Q are elucidated via HPLC method in this study. HPLC analysis data showed that peaks of phthalic acid, catechol, and phenanthrene appeared at a retention time of 1.65, 1.47, and 3.91 min, respectively (Fig. 4A, 4B, and 4C). After three-day cultivation of strain LZ-Q in BH/PHN, the peak at 3.91 min decreased and two main peaks occurred at 1.65 and 1.47 min (Fig. 4D), suggesting that phthalic acid and catechol were generated which is consistent with the previous result that strain LZ-Q could degrade salicylic acid, catechol, and phthalic acid. These results revealed that there are two PHN possible degradation pathways for LZ-Q (inset of Fig. 4D).

**Table 2:** The diversity of degradable substrates by strain LZ-Q

Substrates	Growth situations	Substrates	Growth situations
Phenanthrene	++	Diesel	++
Naphthalene	++	Salicylic acid	+
Anthracene	++	Phthalic acid	++
Pyrene	++	Diphenylamine	+
Petrol	+		

+: Moderate, ++: good

**Fig. 5:**
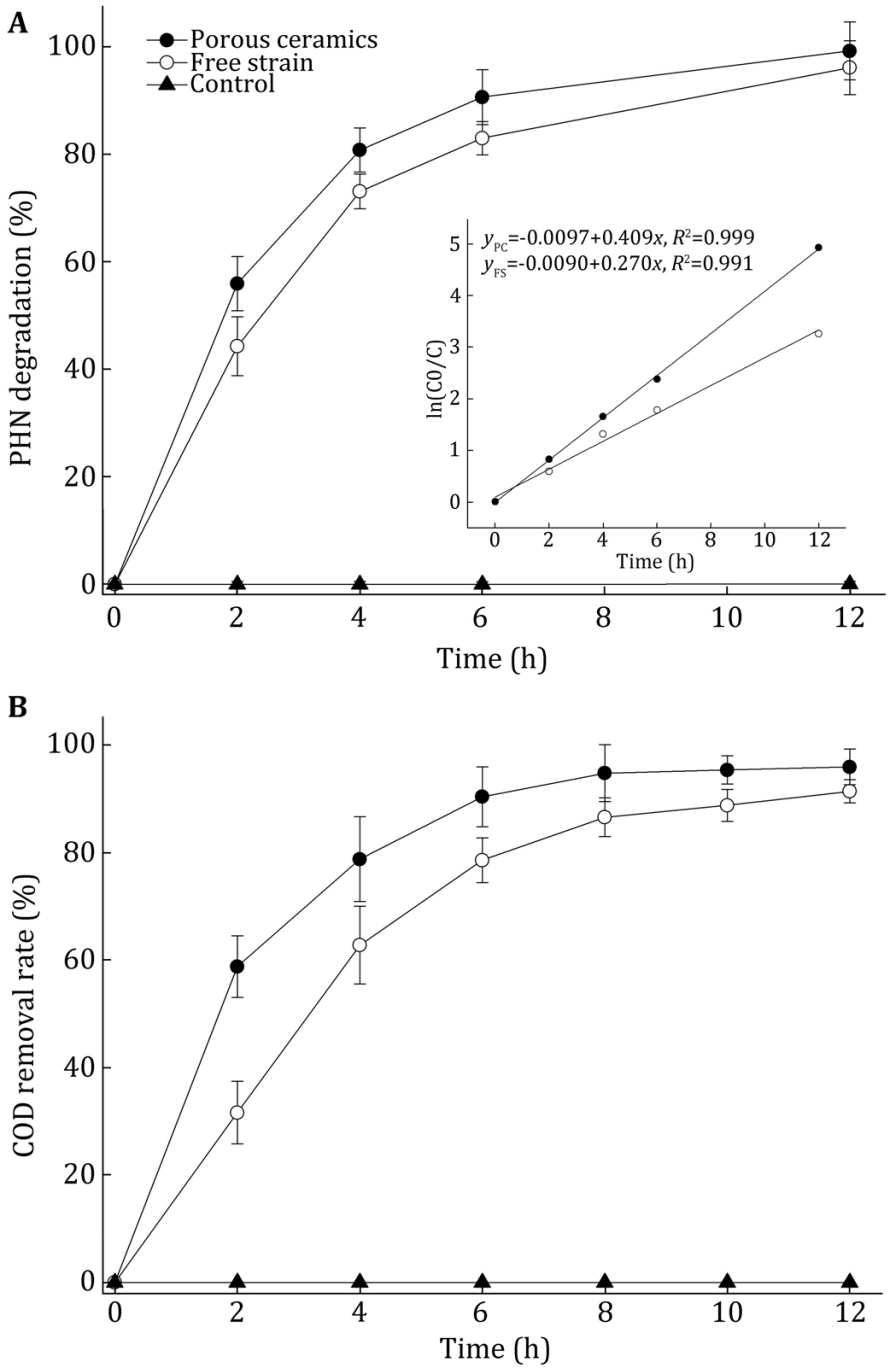
PHN degradation curve (**A**) and COD removal rate curve (**B**) of strain LZ-Q using in MBR. Reactor without strains was used as a control

Studies of metabolic variations of phenanthrene biodegradation have been proved by HPLC method, such as studies of *Pseudomonas putida* NCIB 9816, *Mycobacterium* sp., and *Burkholderia* sp. strain BC1 (Boldrin et al. 1993; Yang et al. 1994; Chowdhury et al. 2014). Among *Pseudomonas* genus, studies about metabolic pathways of phenanthrene degradation showed a variation. *Pseudomonas* strain BZ-3 degraded PHN through salicylic acid pathway (Lin et al. 2014). According to the HPLC result, dimethylphthalate was detected as the intermediate product during PHN degradation. Therefore, *Pseudomonas* sp. USTB-RU biodegrade PHN via the phthalic acid pathway (Masakorala et al. 2013). In this study, strain LZ-Q degraded PHN both via salicylic acid pathway and phthalic acid pathway. This result is in agreement with the previous work revealing that *Pseudomonas* sp. N7 can degrade PHN via both pathways (Jia et al. 2008).

### Immobilized strain LZ-Q in MBR degrades PHN continuously and efficiently under hypersaline and hyperalkaline conditions

Our previous results suggest that LZ-Q is suitable for application in hypersaline and hyperalkaline wastewater treatment. An MBR system was setup to test LZ-Q’s ability to degrade PHN in wastewater. In our study, ceramics are used as adsorbing carriers. Results show that strain LZ-Q grew well on the surface and in the ostioles of ceramics. In order to determine the degradation ability of strain LZ-Q in MBR, microorganisms were treated with synthetic wastewater with 20 mg/L phenanthrene (pH 8 and 35 g/L NaCl). The PHN degradation and COD removal were detected in free-bacteria reactors (FBR) and immobilized-microorganisms reactors (IMR). Decomposition rates of COD and PHN were faster in IMRs than in FBRs. The PHN degradation rate reached 90.68% in IMRs at 6 h, while it was only 82.04% in FBRs with no significant differences (*P* > 0.05). A COD removal rate of 90.4% was achieved when using ceramics as carriers, whereas it was only 78.6% by free strain (Fig. 5). COD removal rate in IMRs had significant difference as compared with that in FBRs (*P* < 0.05). These results reveal that ceramic carriers with strain LZ-Q spur COD removal, suggesting that immobilized technique is suitable for wastewater treatment and similar to report about immobilizing bacteria onto ceramic carriers could remove COD more efficiently during operation (Kariminiaae-Hamedaani et al. 2003). Parameswarappa reported that ceramic material was a good choice for wastewater treatment and immobilization technology enhanced the degrading efficiency of ethylbenzene by *Pseudomonasfluorescens*-CS2 (Parameswarappa et al. 2008). In the batch and semi-continuous treatments, ceramics with immobilized consortia could remove COD, phosphate, nitrate, and H_2_S effectively with removal rates of 89%, 77%, 99%, and 99.8% for 1 month (Nagadomi et al. 2000). In a packed bed bioreactor, 82% of the influent COD was removed within 160 days of operation (Kariminiaae-Hamedaani et al. 2003).

In addition, degradation of PHN in both FBRs and IMRs fitted to the exponential function and followed a pseudo-first-order kinetic model with rate constants of 0.372/h (*R*^2^ = 0.999) and 0.290/h (*R*^2^ = 0.991), respectively (inset of Fig. 5A), and agrees with previous studies on PAHs biodegradation, such as *Pseudomonas aeruginosa* strain PAH-1 whose PHN degradation characteristics fitted to pseudo-first-order kinetic model (Ma et al. 2011).

**Fig. 6:**
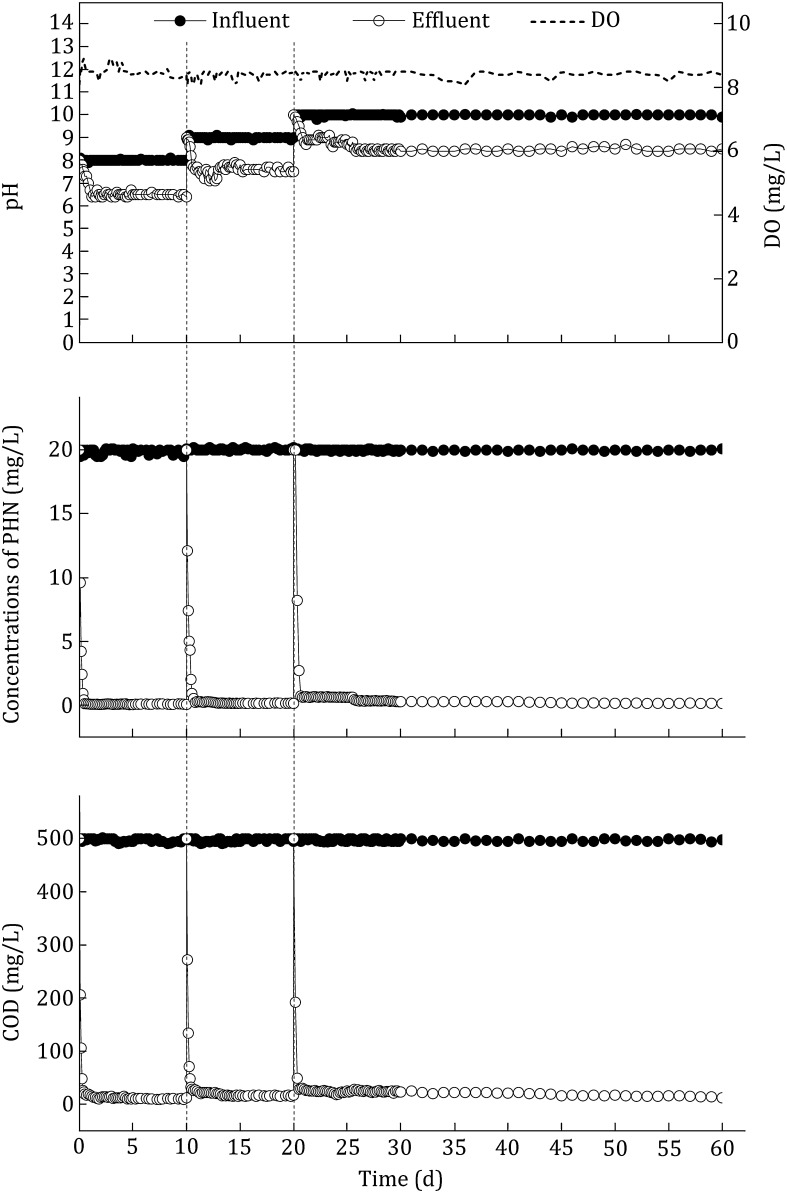
Changes in COD and PHN concentrations of the influent and effluent in the MBR with different pH

The MBRs were operated at 19–21 °C with a HRT of 3 h, and influent COD and PHN concentrations were maintained to 500 and 20 mg/L, respectively. Based on different pHs (8, 9 and 10) of influent, process was divided into three phases. In the first phase, the effluent concentrations of COD and PHN were reduced to 26 and 0.9 mg/L in 8 h. At the 12th hour of the second phase, the COD removal rate of 94.4% and the PHN degradation rate of 95.4% were achieved. In the final phase, the COD removal rate reached 94.3% at the 12th hour, and concentration of PHN dropped to 0.7 mg/L with the degradation rate of 96.5% at the 16th hour. Effluent concentrations of COD and PHN were maintained around 30 and 0.8 mg/L in all three phases (Fig. 6). The MBRs used in this study operated stably for 60 days and degradation ability showed no signs of decreasing.

Membrane bioreactor is a good system to treat industrial wastewater (Melin et al. 2006). It has been reported that MBR could remove more than 94%–98% COD and TOC (Scholz and Fuchs 2000). Cirja reported removal of organic micropollutants by microorganism in MBR (Cirja et al. 2008). *Streptomyces* sp. QWE-35 degraded 200 mg/L naphthalene in a MBR and could be a potential candidate for coal gasification wastewater treatment (Xu et al. 2014). However, in industrial operations of MBR, using free strain to treat wastewater is not efficient, as free strains are hard to separate from aqueous solution and cause membrane fouling (Zhang et al. 2011). Membrane fouling still limits the widespread application of MBR (Tang et al. 2010). Immobilized microorganism technology is a solution for these disadvantages (Xu et al. 2012). Ceramics is a kind of carrier which can decrease the membrane fouling, enhance the removal rate, harbor the long-term usability in wastewater treatment (Kariminiaae-Hamedaani et al. 2003; Bai et al. 2009). In our study, the system immobilized strain LZ-Q onto ceramic carriers in membrane-bioreactor was able to remove more than 96% of the influent phenanthrene and 94% of the influent COD during 60 days of operation when the HRT was 3 h, and degradation ability showed no signs of decreasing.

Hypersaline and hyperalkaline conditions are also limitations of efficiency of wastewater treatment in MBR as biological treatment is strongly inhibited by salts (mainly NaCl) (Lefebvre and Moletta 2006). Soltani reported that microorganisms only degraded 50% PHN in MBR system under high salinity conditions (Soltani et al. 2010). When the salt concentration was up to 84 mg/L, MBR system requires 73 operating days adaptation to recover to the normal COD removal efficiency (Artiga et al. 2008). However, our data indicated that strain LZ-Q could degrade PAHs including phenanthrene efficiently under the hyperhaline and hyperalkaline conditions. The MBR system with immobilized strain LZ-Q operated stably for 60 days without decreasing. All these results suggesting immobilized strain LZ-Q in MBR is a candidate system for degrading phenanthrene in saline-alkali wastewater.

## CONCLUSIONS

An efficient phenanthrene degradation strain LZ-Q, which was isolated from petroleum-contaminated soil in Lanzhou reach of the Yellow River, was identified as *P.* sp. by ViTek 2 and 16S rRNA gene sequencing. The antibiotic-sensitive strain LZ-Q grew in BH medium containing 1 g/L phenanthrene as the carbon source and had the ability to degrade phenanthrene with a broad range of salinities (5–75 g/L), pHs (5–10), and temperatures (10–42 °C). Strain LZ-Q degraded phenanthrene efficiently through salicylic acid pathway and phthalic acid pathway. In the BH/PHN (100 mg/L) medium, the PHN degradation rate of strain LZ-Q was 92.27% in 5 days. In addition, strain LZ-Q could use other organic compounds as carbon sources, such as naphthalene, anthracene, pyrene, petrol, and diesel. Compared with free strain, adsorptive ceramic carrier of strain LZ-Q spurs removal rates of COD and PHN from 78.6% to 90.4% and 82.04% to 90.68% at 6 h. Continuous treatment performance of MBR with immobilized strain LZ-Q operated powerfully and stably in this study. All the observations indicated that MBR with immobilized *P.* sp. LZ-Q could be a candidate to remediate phenanthrene-contaminated saline-alkali sewage.

## MATERIALS AND METHODS

### Sample collection

Soil sampling was carried out near the sewage outlet of a petrochemical company (36°02′N, 103°61′E, Lanzhou, China) in Gansu province, which is in upper reach of the Yellow River in April, 2013. The climate of sampling site is a typical temperate and monsoonal continental climate, with an annual mean air temperature of 9.3 °C and precipitation of 360 mm (Wu et al. 2011). Samples were collected at depth of 15 cm at 22 °C and pH 6.2. After collection, samples were immediately transferred to the laboratory in sterilized aluminum boxes and stored at −80 °C.

### Reagents and culture media

Phenanthrene, salicylic acid, catechol, phthalic acid, and methyl alcohol were HLC grade, and all the other chemicals were of analytical grade.

Bushnell-Hass mineral salt medium (BH) was composed of (g/L) NaCl 5, KH_2_PO_4_ 1, K_2_HPO_4_ 1, NH_4_NO_3_ 1, MgSO_4_·7H_2_O 0.2, CaCl_2_·2H_2_O 0.02, and FeCl_3_ 0.05. Luria–Bertani (LB) medium was composed of (g/L) peptone 10, NaCl 5, and yeast extract 10. Per liter solid LB contained 15 g agar powder. One liter of synthetic wastewater (SW) consisted of NaCl 35 g, glucose 450 mg, FeCl_2_·4H_2_O 200 mg, CaCl_2_·2H_2_O 200 mg, MgCl_2_·6H_2_O 300 mg, CuCl_2_·2H_2_O 0.6 mg, ZnCl_2_ 1 mg, H_3_BO_3_ 1 mg, MnCl_2_·4H_2_O 10 mg, CoCl_2_·6H_2_O 1 mg, NaMoO_4_·2H_2_O 0.2 mg, NiCl_2_·6H_2_O 1 mg. NH_4_Cl, and K_2_HPO_4_ were added into the SW to maintain COD:N:P ratio of 200:5:1. The pH of the SW was adjusted with 25 g/L NaHCO_3_. All prepared media were autoclaved at 121 °C for 20 min.

### Enrichment and isolation of phenanthrene-degrading strain

Phenanthrene-degrading strains were isolated from the mixed soil samples taken from Lanzhou reach of Yellow River. 1 g soil was added into 100 mL BH supplied with 50 mg phenanthrene as sole carbon and energy source. Enrichments were incubated at 28 °C and 180 r/min. After 5 days, 1 mL of enriched aqueous culture was transferred to another BH/phenanthrene (500 mg/L) medium. Consecutive enrichment processes were repeated three times for every 5 days until microbial consortium was developed in the medium. Then, 100 μL dilute aqueous culture was spread on the solid BH plate. After 2 days, developed colonies on the plates were isolated and inoculated into the BH/phenanthrene medium to confirm their potential of degrading phenanthrene. Repeated plate streaking was employed to ensure purity of the isolates. A mixture of the bacterial solution and 50% glycerol with a ratio of 1:1 (*v*/*v*) were stored at −20 °C.

### Characterization and identification of microorganism

The isolated bacteria were examined using observation of morphological features. Gram staining was performed before biochemical tests were done with the Vitek 2 System (bioMerieux Industry, Marcyl’Etoile, France) according to the manufacturer’s instructions.

Molecular identification was carried out by phylogenetic analysis following the 16S rRNA sequencing. The strain was first activated in 5 mL LB on a shaker at 30 °C and 180 r/min for 12 h. Amplification of gene fragments encoding 16S rRNA was performed using the universal 16S rRNA primers (*E. coli* 27F and 1492R). Fragments sequencing was done by Shanghai Majorbio Bio-pharm Technology Co. Ltd (Shanghai, China) and sequences were analyzed at EzTaxon (www.eztaxon.org/) database. Phylogenetic tree was generated by MEGA (Tamura et al. 2007).

### Determination of growth characterization and degradation ability

#### Growth on phenanthrene

The optimum temperature test was carried out under the temperatures of 10, 28, 37, and 42 °C. Optimum pH was tested at pH 5, 6, 7, 8, 9, and 10. Growth curves were determined by measuring *OD*_600nm_ in the medium using a spectrophotometer. The isolated strain was separately inoculated into the 100 mL sterilized BH medium with 100 mg/L phenanthrene as sole carbon and cultivated at pH 7, 180 r/min, and 28 °C. *Escherichia coli* was used as negative control.

#### Growth on other carbon sources

Growth of the pure culture on other carbon substrates, including naphthalene, anthracene, pyrene, salicylic acid, phthalic acid, diphenylamine, petrol, and diesel was tested to find out the potential degradable substrate as sole carbon and energy source. All experiments were in triplicate.

#### Degradation of phenanthrene

1 mL aliquots of isolated strain were added into 100 mL sterilized BH medium containing 100 mg/L phenanthrene for 5 days. Quantity of phenanthrene concentrations and varieties of catabolic intermediates were determined using HPLC. Bacterial solutions were filtered through disposable filters (0.45 μm). Phenanthrene and putative metabolites were separated with a silica C18 column (4.6 × 150 mm). The mobile phase consisted of 80% methanol and 20% water at a flow rate of 1 mL/min and room temperature. Eluants were monitored by UV-Vis light detection at a wavelength of 254 nm and qualified using an external standard calibration curve.

#### Tolerance of salinity and antibiotics

Salinity tolerance of the isolate was assayed on BH/PHN(100 mg/L) liquid containing 5, 35, 40, 50, 75, and 100 g/L NaCl. The *OD*_600nm_ was reported in 120 h of incubation. Antibiotic sensitivity tests were carried out by the Kirby-Bauer antibiotic susceptibility disk diffusion method (Kirby et al. 1966). All experiments were in triplicate.

### Bacteria immobilization and reactor setup

Ceramic-microorganism adsorptive carriers were spherical in shape with the diameter of 2–3 mm, and were prepared as follows: washing with water for three times, and then immersed in 5% HCl, neutral water, and 5% NaOH for 2 h successively. 100 mL modified ceramics was placed into 500-mL flasks containing 200 mL bacterial culture for 3 days with changing LB liquid medium every 24 h. The obtained carriers were washed with normal saline and stored in physiological saline at 4 °C before using.

**Fig. 7:**
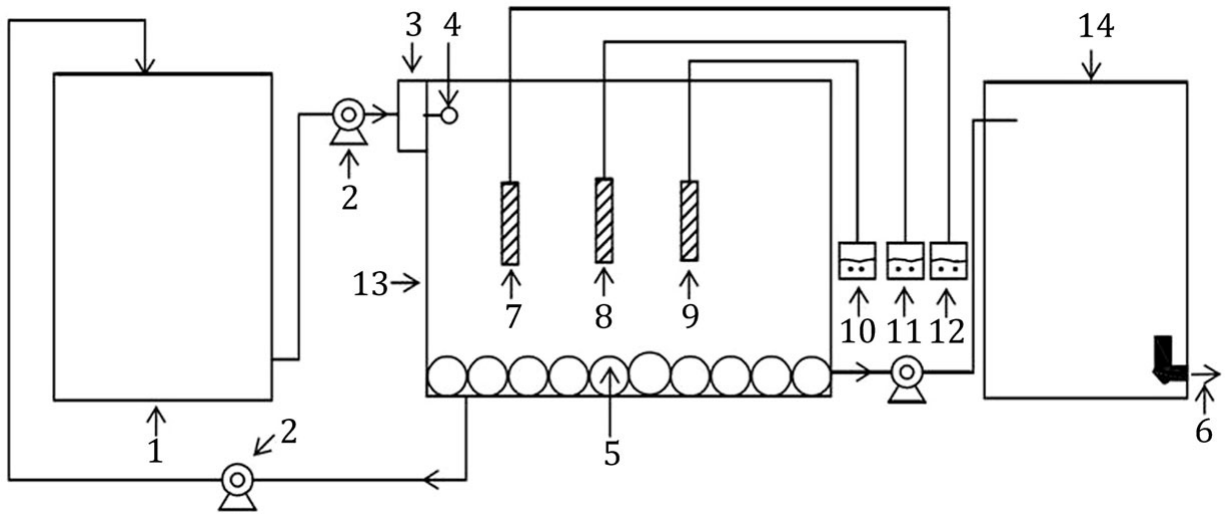
Simplified scheme of the immobilized microorganisms reactor. *1*. Sewage storage tank; *2*. Suction pump; *3*. Rotameter; *4*. Inlet; *5*. Aerator; *6*. Outlet; *7*. DO sensor; *8*. Temperature sensor; *9*. pH sensor; *10*. pH monitor; *11*. Temperature monitor; *12*. DO monitor; *13*. Aeration tank; *14*. Membrane reactor

This research was carried out in continuous aerating MBRs with 2 L (3 L of total volume) (Fig. 7). The MBRs operated with micro-filtration (MF) flat-sheet (FS) membrane module with nominal porosity of 0.4 μm. Free-bacteria or immobilized microorganism carriers were added into reactors. All the other operating conditions for reactors were kept the same. MBRs were operated at 19–21 °C and were run at 8–12 mg/L DO with the help of an aeration pump. Hydraulic retention time (HRT) was 3 h. The experiment lasted for 60 days and each reactor was set up in triplicate.

### Morphological observations

Morphological observation of scanning electron microscopy (SEM) was done with Hitachi S-3400 N Scanning Electron Microscope (Hitachi High-Technologies Corporation, Tokyo, Japan). Sample preparation for SEM was carried out according to the methods reported before (Zhou et al. 2009).

### Chemical analysis

The chemical oxygen demand (COD_Cr_) was determined by the standard method based on potassium dichromate (K_2_Cr_2_O_7_) oxidization method (Zhou et al. 2009).
